# Every Man a Landed Proprietor

**Published:** 1855-09

**Authors:** A. W. Worthington


### EVERY MAN A LANDED PROPRIETOR.

By the Rev. A. W. Worthington, B.A.

Freehold Land Societies, first established for a political purpose, are working in other ways than was originally intended. Many, who subscribed to them for the sake of a qualification to vote, have neglected to put their names on the register; because they do not like to possess a privilege by the free use of which they might offend their employers, and thus injure their prospects of advancement, or lose their situations. The allotments, too, have frequently changed hands, because the original subscribers could not pay up the calls. Speculators will purchase a number of lots, and build slight and unhealthy cottages upon them; or lend money for that purpose to the original proprietors on mortgage of the land, by the foreclosing of which they may become possessed of the property at a comparatively low price. The cottages are too often built without the assistance of an architect, in utter ignorance and disregard of the simplest laws of health, and without regard for convenience or beauty.

The management of the estates has at times fallen into hands utterly incapable of discharging the duties; and I have heard one case in which the treasurer decamped with the funds of the society in his hands.

It not unfrequently happens that, when an estate has been purchased, no further arrangement of it is made than to stake out the streets and distribute the allotments. This is done frequently without any reference to the proper fall for drainage, or other matters necessary for convenience and health. No rules are laid down for the building of houses; and thus one cottage is built close to the road; another at the extreme end of the allotment, and close, it may be, to the pigsty in the neighbouring garden. One cottage

---

"When they are dead, upon their tomb is seen,
Not what they were, but what they should have been."

faces the street; another turns its back or side upon it, while some stand cornerwise, as though they had all been sown broadcast upon the so-called California.

The society at Lancaster has managed its estate much better. The managers laid out the roads with a due regard to fall for drainage, paved the streets, and made the main sewers with proper arrangements for attaching house-drains to them when necessary. The extra expense thus incurred was divided among the allotments, to the cost-price of which it added little, while a security was gained for the health and convenience of the estate which could probably never have been arrived at in any other way.

The importance of such an arrangement is at once evident, as well as the advantage to be derived by making due provision for the uniform position of the houses, and from giving to each allottee a printed list of simple directions as to the best mode of building a cheap, convenient, elegant, and, above all, a healthy house. I am acquainted with an architect (and would recommend his plan for imitation) who, for a moderate fee, revises the plans of cottage houses after they have been designed by the person about to build.

But the freehold land societies, though subject to the abovementioned evils, are working beneficially in other respects. The possession of a landed estate, even though small, gives a man self-respect. Having, as it has been called, "a stake in the country," he begins to take a *sober* interest in politics, and is less likely to be carried away by the flighty doctrines of stump-orators or socialistic publications. If he can build a cottage, he probably becomes a family-man; and he himself, as well as his wife and children, take that care to preserve and improve the house and garden which is so rarely bestowed by a tenant on the property of his landlord. If he should not wish to build, he will probably turn his plot of land into a garden, and thus have a healthy and interesting employment which will provide him food in the shape of vegetables, simple medicines in the form of savoury herbs, and, if he turn his attention to bees, will afford him a new field of interesting study and profitable employment. His moral character will be thus improved: for instance, I have heard of one case where a notorious drunkard was led from the public-house by the pleasure of gardening in his allotment in a freehold land society. The mind is opened to religious impressions; for he sees, enjoys, admires, and by degrees begins to study the beauties and wonders of nature.

In this and many other ways, I believe that the increase in number of small proprietors of land, whether by freehold land societies, by the aid of intelligent employers of labour, or by independent purchase on the part of working men, may lead to important social and moral results, as it will increase in the country a class of simple, earnest, healthy, hearty and intelligent men, resembling, though in many respects superior to the stalwart yeomen, who exercised so great an influence in earlier periods of our history.

